# Complete genome sequence of *Lutibacter profundi* LP1^T^ isolated from an Arctic deep-sea hydrothermal vent system

**DOI:** 10.1186/s40793-016-0219-x

**Published:** 2017-01-07

**Authors:** Juliane Wissuwa, Sven Le Moine Bauer, Ida Helene Steen, Runar Stokke

**Affiliations:** 1 Centre for Geobiology, University of Bergen, Bergen, Norway; 2 Department of Biology, University of Bergen, Bergen, Norway

**Keywords:** *Lutibacter*, *Flavobacteriaceae*, Loki’s castle, Biofilm, Deep-sea hydrothermal vent

## Abstract

*Lutibacter profundi* LP1^T^ within the family *Flavobacteriaceae* was isolated from a biofilm growing on the surface of a black smoker chimney at the Loki’s Castle vent field, located on the Arctic Mid-Ocean Ridge. The complete genome of *L. profundi* LP1^T^ is the first genome to be published within the genus *Lutibacter. L. profundi* LP1^T^ consists of a single 2,966,978 bp circular chromosome with a GC content of 29.8%. The genome comprises 2,537 protein-coding genes, 40 tRNA species and 2 rRNA operons. The microaerophilic, organotrophic isolate contains genes for all central carbohydrate metabolic pathways. However, genes for the oxidative branch of the pentose-phosphate-pathway, the glyoxylate shunt of the tricarboxylic acid cycle and the ATP citrate lyase for reverse TCA are not present. *L. profundi* LP1^T^ utilizes starch, sucrose and diverse proteinous carbon sources. In accordance, the genome harbours 130 proteases and 104 carbohydrate-active enzymes, indicating a specialization in degrading organic matter. Among a small arsenal of 24 glycosyl hydrolases, which offer the possibility to hydrolyse diverse poly- and oligosaccharides, a starch utilization cluster was identified. Furthermore, a variety of enzymes may be secreted via T9SS and contribute to the hydrolytic variety of the microorganism. Genes for gliding motility are present, which may enable the bacteria to move within the biofilm. A substantial number of genes encoding for extracellular polysaccharide synthesis pathways, curli fibres and attachment to surfaces could mediate adhesion in the biofilm and may contribute to the biofilm formation. In addition to aerobic respiration, the complete denitrification pathway and genes for sulphide oxidation e.g. sulphide:quinone reductase are present in the genome. sulphide:quinone reductase and denitrification may serve as detoxification systems allowing *L. profundi* LP1^T^ to thrive in a sulphide and nitrate enriched environment. The information gained from the genome gives a greater insight in the functional role of *L. profundi* LP1^T^ in the biofilm and its adaption strategy in an extreme environment.

**Electronic supplementary material:**

The online version of this article (doi:10.1186/s40793-016-0219-x) contains supplementary material, which is available to authorized users.

## Introduction

The type strain *Lutibacter profundi* LP1^T^ (=DSM 100437 ^T^ =JCM 30585 ^T^) belongs to the family *Flavobacteriaceae* within the phylum *Bacteroidetes* [1]. Members of this family are abundant in marine and freshwater habitats and have been isolated from seawater [2, 3], sea ice [4], fresh water [5], glaciers [6, 7], marine plants and animals [4, 8]. In addition, metagenomic studies have shown the presence of *Bacteroidetes* in marine sediments [9, 10]. Members of the *Flavobacteriaceae* are also found in the human microbiota [11, 12], soil [13], insects [14], food and dairy products [15]. The family *Flavobacteriaceae* has been proposed to play an important role in the degradation of organic matter and nutrition turnover in the oceans [16]. They have been identified either as free-living or attached to organic detritus particles and phytoplankton in marine surfaces [17, 18] and in deep-sea planktonic communities [19]. Biopolymers, such as cellulose, chitin and proteins are part of the high molecular mass fraction of (dissolved) organic material in aquatic habitats. The ability to degrade such polymers has been shown for *Flavobacteriaceae* in both culture-dependent and independent studies [16, 20]. A multiplicity of strains has been isolated and several genomes sequenced [21–23]. Genomic analyses of marine isolates have revealed a large number of GHs, GTs, peptidases and adhesion proteins, as well as genes for gliding motility, supporting an organotrophic life style as HMW organic matter degraders [21–24].

In 2006 the first *Lutibacter* strain, *L. litoralis* CL-TF09^T^, was isolated and introduced as a new organotrophic genus of the *Flavobacteriaceae* family [25]. Until now, all published strains have been isolated from Korean coastal waters or the Sea of Japan, and found either as free-living or in association with invertebrates [3, 25–31]. In contrast, *L. profundi* LP1^T^ was isolated from a biofilm attached to the outer surface of a black smoker chimney from the LCVF located at the AMOR [1]. In the biofilm, the *Bacteroidetes* population was attached as ectobionts on the outer surface of filamentous *Epsilonproteobacteria* [32]. Here we present the complete genome of *Lutibacter profundi* LP1^T^, the first genome to be published from the genus *Lutibacter*. The genomic features of *L. profundi* are presented and its possible role in the biofilm community and its biotechnological potential is discussed.

## Organism Information

The isolation and characterization of *L. profundi* LP1^T^ has previously been described [1]. Thus, the organism information will be given as a short summary supplemented with additional information.

### Classification and features

*L. profundi* LP1^T^ was isolated from a biofilm attached to the surface of a black smoker chimney wall at the LCVF, on the AMOR [32–34]. A steep temperature gradient between the up to 320°C hydrothermal fluids and the −0.7°C cold surrounding seawater places the biofilm in a mesophilic temperature range [33, 35]. Artificial seawater medium [36] supplemented with modified Wolfe’s mineral solution without NaCl or CaCl_2_ (0.001%), Wolfe’s vitamin solution (0.5%), 10mM Na_2_S and yeast extract (0.01%) under microaerobic conditions was used for primary enrichments and isolation of *L. profundi* LP1^T^ [1].

**Fig. 1:**
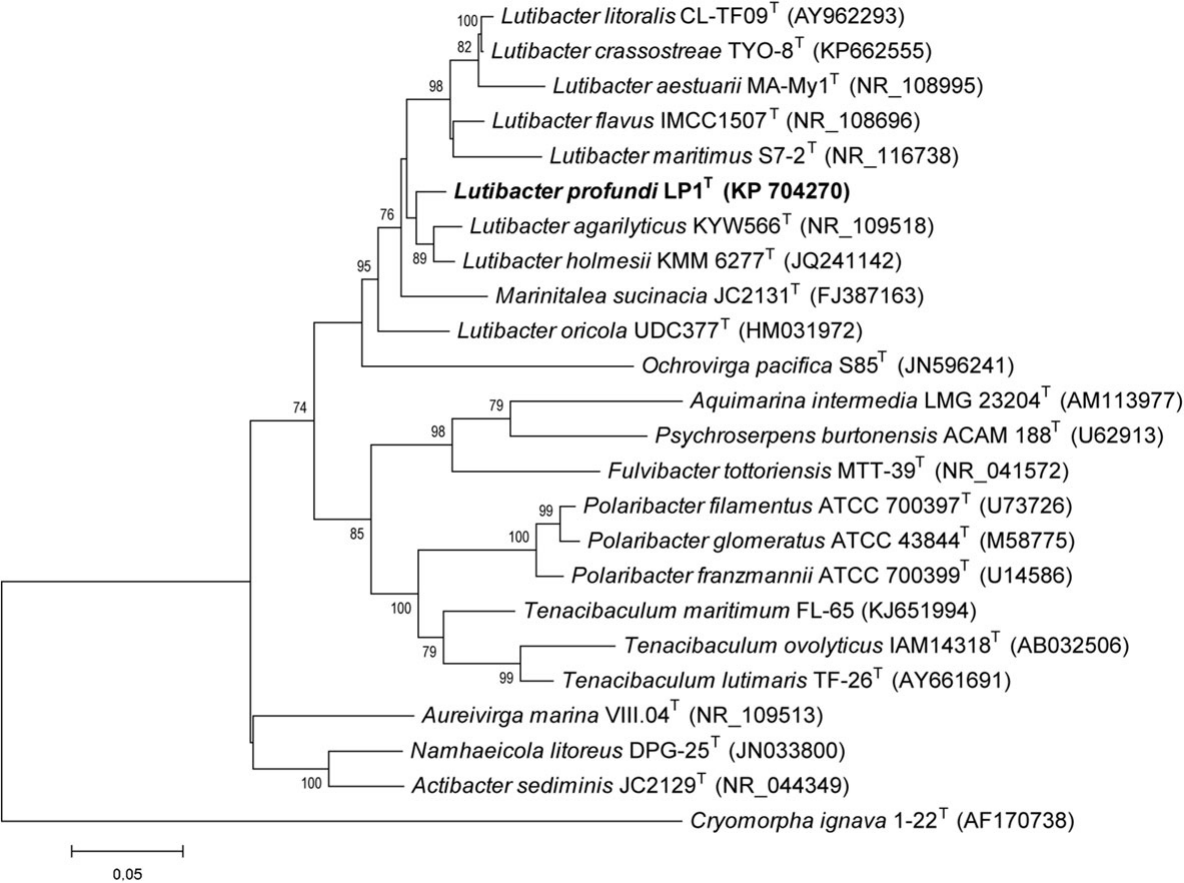
Phylogenetic tree displays the position of *Lutibacter profundi* LP1^T^ (shown in bold) relative to the other type strains of *Lutibacter* based on 16S rRNA. The phylogenetic tree was generated after trimming the alignment using MUSCLE [82, 83] to 1323 aligned positions, using maximum likelihood method with general time reversible model as preferred model incorporated in MEGA v. 6.06 [84]. At the branch points bootstrap values above 70, expressed as percentage of 1000 replicates, are shown. Bar: 0.05 substitutions per nucleotide position. *Cryomorpha ignava* 1-22^T^ (AF170738) was used as outgroup

The genus *Lutibacter* *,* including *L. profundi* LP1^T^, thus far comprise nine strains which are proposed to represent novel species: *L. litoralis* CL-TF09^T^ [25], *L. maritimus* S7-2^T^ [26], *L. aestuarii* MA-My1^T^ [27], *L. flavus* ^T^ [29]*,* *L. agarilyticus* KYW566^T^ [28], *L. oricola* UDC377^T^ [3], *L. crassostreae* TYO-8^T^ [30] and *L. holmesii* KMM 6277 ^T^ [31]. The strain *L. crassostreae* TYO-8^T^ was isolated from an oyster collected from the South Sea, South Korea [30], whereas *L. holmesii* KMM 6277 ^T^ was isolated from an sea urchin collected from Troitas Bay, Sea of Japan [31]. The other species were isolated from shallow coastal waters or tidal areas around the coast of South Korea [3, 25–29]. So far, *L. profundi* LP1^T^ is the only *Lutibacter* strain isolated outside of South Korean Territory. *L. profundi* LP1^T^ shared between 94.7% (*L. maritimus* S7-2^T^) and 97.5% (*L. holmesii* KMM 6277 ^T^) 16S rRNA gene identity with the other *Lutibacter* strains. 16S rRNA phylogenetic analysis placed strain LP1^T^ closest to *L. agarilyticus* KYW566^T^ and *L. holmesii* KMM 6277 ^T^ within the *Lutibacter* group, as previously described (Fig. 1) [1].

**Fig. 2:**
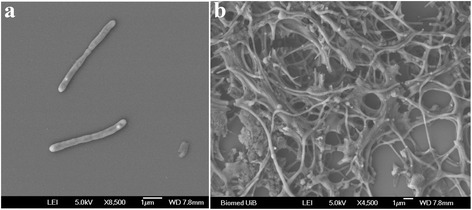
Scanning electron microscopy of *Lutibacter profundi* LP1^T^. **a** Normal cultivation conditions, **b** Oxygen stress (atmospheric oxygen level)

*L. profundi* LP1^T^ was described as Gram-negative, microaerophilic, non-motile rods [1] (Fig. 2a). *L. profundi* LP1^T^ grew in a temperature range between 13 and 34 °C with an optimum of 23 °C, a pH range between 5.5 and 7.5 with pH 6–6.5 as optimum [1]. *L. profundi* LP1^T^ grew in NaCl concentrations ranging from 1 to 3%, with an optimal concentration of 2% (Table 1). However, the strain was not able to grow with NaCl as the sole source of salt. No growth was observed under fermentative or anaerobic conditions using NO_3_^−^ and S_2_O_3_^2−^ as electron acceptors. Nevertheless, nitrate was reduced to nitrite under anaerobic and microaerophilic conditions. *L. profundi* LP1^T^ was tested positive for oxidase and catalase activity [1]. Using the API ZYM system (BioMérieux, France), *L. profundi* LP1^T^ showed strong activity for alkaline phosphatase, leucine arylamidase, valine arylamidase, trypsin, acid phosphatase, naphtol-AS-BI-phosphohydrolase and N-acetyl-Beta-glucosaminidase, as well as weak activity for esterase lipase and alpha-glucosidase. Following carbon sources were utilized in an AN microplate™ (Biolog, USA) test: pyruvic acid, L-alanyl-L-glutamine, L-alanyl-L-threonine, L-glutamic acid, glycyl-L-proline and L-threonine, in addition to L-proline, L-glutamate and pyruvate with 0.01% extra YE [1]. Furthermore, *L. profundi* LP1^T^ was able to grow on D-sucrose supplemented with 0.01% yeast extract, but not on D-glucose, D-fructose, D-cellobiose and D-galactose. *L. profundi* LP1^T^ did not utilize glycerol, citrate, succinate, L-leucine and tartrate supplemented with yeast extract [1]. *L. profundi* LP1^T^ was able to hydrolyse gelatin, casein, starch and indoxyl acetate, but not agar, cellulose, urea, esculine, lecithin, tween 80 or tween 20. Cells were resistant to streptomycin, however susceptible to ampicillin, penicillin, erythromycin, tetracyclin and chloramphenicol.

**Table 1:** Classification and general features of *Lutibacter profundi* LP1^T^ according to MIGS standards [39]

MIGS ID	Property	Term	Evidence code
	Classification	Domain *Bacteria*	TAS [75]
		Phylum *Bacteroidetes*	TAS [76, 77]
		Class *Flavobacteriia*	TAS [78]
		Order *Flavobacteriales*	TAS [77, 79]
		Family *Flavobacteriaceae*	TAS [80]
		Genus *Lutibacter*	TAS [25, 27]
		Species *Lutibacter profundi*	TAS [1]
		Type strain: LP1 (DSMZ 100437^T^ = ^T^)	TAS [1]
	Gram stain	Gram-negative	TAS [1]
	Cell shape	Rod	TAS [1]
	Motility	Non-motile	TAS [1]
	Sporulation	no	TAS [1]
	Temperature range	13–34°C	TAS [1]
	Optimum Temperature	23°C	TAS [1]
	pH range; optimum	5.2–7.5; 6.2	TAS [1]
	Carbon sources	tryptone	TAS [1]
MIGS-6	Habitat	Marine, biofilm attached to black smoker chimney	TAS [1]
MIGS-6.3	Salinity	1–3%	TAS [1]
MIGS-22	Oxygen requirement	Microaerobic, aerobic	TAS [1]
MIGS-15	Biotic relationship	Free-living	TAS [1]
MIGS-14	Pathogenicity	Non-pathogen	NAS
MIGS-4	Geographic location	Loki’s Castle, Arctic mid-Ocean ridge	TAS [33]
MIGS-5	Sample collection	Summer 2009	TAS [32, 34]
MIGS-4.1	Latitude	73.33.97N,	TAS [1, 32, 34]
MIGS-4.2	Longitude	08.09.51E	TAS [1, 32, 34]
MIGS-4.4	Altitude	−2350m	TAS [1, 32, 34]

Evidence codes – *IDA* inferred from direct assay, *TAS* traceable author statement (i.e., a direct report exists in the literature), *NAS* non-traceable author statement (i.e., not directly observed for the living, isolated sample, but based on a generally accepted property for the species, or anecdotal evidence). These evidence codes are from the Gene Ontology project [81]

In the current study, *L. profundi* LP1^T^ tested negative for the utilization of the following additional carbohydrates; D-maltose, D-mannose, L-arabinose, D-trehalose, D-xylose, D-cellulose and chitin.

#### Chemotaxonomic data

The composition of the major cellular fatty acids in *L. profundi* LP1^T^ varies depending on the used media and growth condition [1]. After growth on marine broth 2216 agar plates the major cellular fatty acids are iso-C_15:0_ (25.2%), iso-C_15:0_ 3-OH (14.5%), iso-C_17:0_ 3-OH (9.6%), iso-C_15:1_ (G) (9.0%), anteiso-C_15:0_ (8.2%), iso-C_16:0_ 3-OH (5.4%) and summed feature I iso-C_15:1_ (H)/C_13:0_ 3OH (7.4%) [1]. The major cellular fatty acid composition varied between the different *Lutibacter* type strains [1]. The major polar lipids of *L. profundi* LP1^T^ are DPG, PE, one unidentified aminolipid and two unidentified lipids, where PE is the main polar lipid. In accordance with the genus, menaquinone-6 (MK-6) is the only respiratory quinone [1].

## Genome sequencing information

### Genome project history

*L. profundi* LP1^T^ as the type strain is the first *Lutibacter* isolate from a deep-sea hydrothermal vent system. The bacterium was chosen for sequencing to study its genomic features in relation to the environmental system it originated from and its biotechnological potential.

Sequencing was conducted at NSC, Norway [37]. Assembly, finishing and polishing steps were performed at the Centre for Geobiology, University of Bergen, Norway. To fulfil NCBI standards the annotation of the genome was performed using the automatic NCBI PGAAP [38]. The complete genome sequence and annotation data of *L. profundi* LP1^T^ is accessible in GenBank under the accession number CP013355. The project information and its association with MIGS version 2.0 compliance [39] have been summarized in Table 2.

**Table 2:** Project information

MIGS ID	Property	Term
MIGS-31	Finishing quality	Finished
MIGS-28	Libraries used	Pacific Biosciences 10 kb library
MIGS-29	Sequencing platform	PacBio
MIGS-31.2	Fold coverage	76x
MIGS-30	Assemblers	Hierarchical Genome Assembly Process (HGAP) v2
MIGS-32	Gene calling method	Prodigal
	Locus Tag	Lupro
	Genbank ID	CP013355
	Genbank Date of Release	February 1., 2016
	GOLD ID	Gp0134121
	BIOPROJECT	PRJNA304382
MIGS-13	Source Material Identifier	DSMZ 100437^T^ =JCM 30585^T^
	Project relevance	Environmental

### Growth conditions and genomic DNA preparation

*L. profundi* LP1^T^ was grown by gently shaking in M1 broth medium at microaerophilic conditions and 23 °C. The high molecular DNA of a 60 ml culture was isolated using a modified method of Marmur [40, 41].

### Genome sequencing and assembly

A 10 kb library was prepared using Pacific Bioscience 10 kb library preparation protocol and BluePippin (Sage Science) for the final size selection. Two SMRT cells were used for sequencing the library on a Pacific Bioscience RS II instrument in combination with the P4-C2 chemistry. In total, 63,994 reads with an average length of 5671 bp were obtained generating a total number of 362.9 Mbp. The raw reads were filtered prior *de novo* assembly using HGAP v2 (Pacific Bioscience) [42], which resulted in one 2,978,418 bp contig with an average coverage of 76.29. Using the Gepard dotplot [43], verified a single highly accurate self-overlapping contig. Minimus2 from the AMOS software package [44] was used to perform the circularization and trimming of the chromosomal contig. Final polishing steps using the RS_Resequencing protocol implemented in the SMRT Analysis software (Pacific Biosciences), resulted in a 2,966,978 bp circular chromosome with a consensus concordance of 99.9%. The location of the dnaA gene was manually relocated and used as start of the chromosome.

### Genome annotation

In order to comply to NCBI standards, the annotation of the genome was performed using the automatic NCBI PGAAP [38]. In addition, SignalP and TMHH-plugins in CLC Genomics Workbench (Qiagen, version 9) was used for the identification of genes with signal peptides and transmembrane helices, respectively.

## Genome properties

**Fig. 3:**
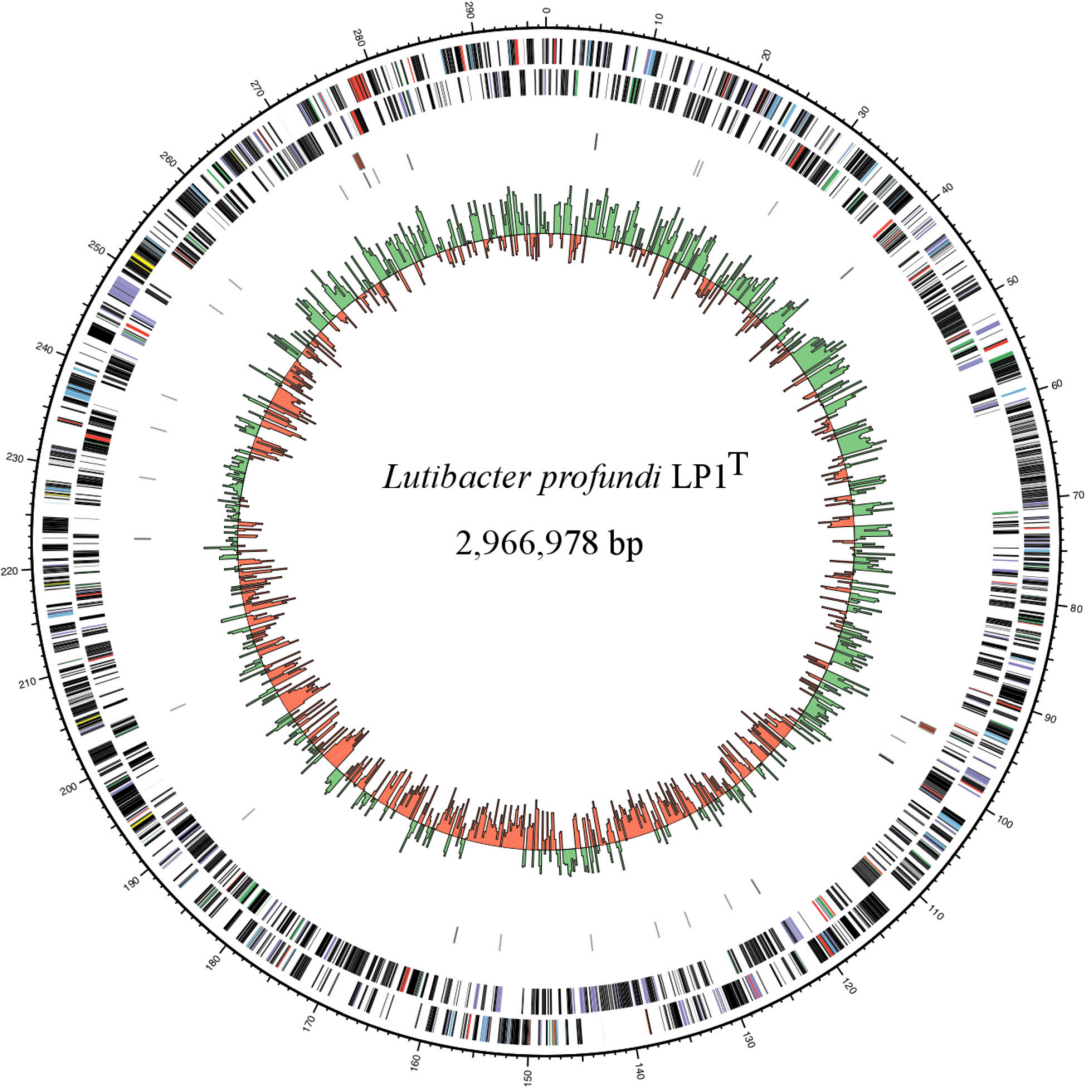
Circular representation of the *Lutibacter profundi* LP1^T^ genome displaying relevant genome features. Circles representing the following (from centre to outside): *1*, G + C skew [(G – C)/(G + C) using a 2-kbp sliding window] (green, positive G + C skew; red, negative G + C skew); *2*, tRNAs (black); *3*, rRNA operons (red); *4*, Coding DNA sequence on the reverse strand; *6*, CDS on the forward strand. Colour coding of CDS was based on COG categories. The figure was build using Circos version. 0.67–6 [45]

The circular genome of *L. profundi* LP1^T^ consists of 2,966,978 bp with a GC content of 29.8%. The chromosome comprises 6 rRNAs located in two operons, 40 tRNAs and one ncRNA (Table 3). The two 16S rRNA genes are identical in DNA sequence. Of 2537 predicted protein-coding genes 1531 were assigned to a putative function and 1006 as hypothetical proteins. In total 96.3% of protein-coding genes were assigned to COG functional categories summarized in Table 4. A Circos [45] genome atlas is presented in Fig. 3. The MEROPS peptidase database [46] and dbCAN [47] were used for identification of peptidases and carbohydrate-degrading enzymes. Identification of conserved domains using the NCBI Batch web CD-Search Tool [48] complemented the analysis. A putative episome of 89 genes is located inside the genome (10.15 kb: 608003-709593) including several plasmid stabilization genes and hypothetical genes.

**Table 3:** Genome statistics

Attribute	Value	Percent of total
Genome size (bp)	2,966,978	100.00
DNA coding (bp)	2,681,332	90.4
DNA G + C (bp)	815,201	27.5
DNA scaffolds	1	
Total genes	2,611	100
Protein coding genes	2,537	97.2
RNA genes	47	1.8
Pseudo genes	27	1
Genes in internal clusters	ND	
Genes with function prediction	1,531	58.6
Genes assigned to COGs	2,447	96.3
Genes with Pfam domains	2,092	82.2
Genes with signal peptides	219	8.4
Genes with transmembrane helices	617	23.6
CRISPR repeats	2

**Table 4:** Number of genes associated with general COG functional categories

Code	Value	Percent age^a^	Description
J	131	5,2	Translation, ribosomal structure and biogenesis
A	0	0	RNA processing and modification
K	88	3,5	Transcription
L	105	4,1	Replication, recombination and repair
B	0	0	Chromatin structure and dynamics
D	17	0,7	Cell cycle control, cell division, chromosome partitioning
V	35	1,4	Defence mechanisms
T	67	2,6	Signal transduction mechanisms
M	160	6,3	Cell wall/membrane/envelope biogenesis
N	3	0,1	Cell motility
U	21	0,8	Intracellular trafficking, secretion, and vesicular transport
O	92	3,6	Posttranslational modification, protein turnover, chaperones
C	161	6,3	Energy production and conversion
G	55	2,2	Carbohydrate transport and metabolism
E	179	7,1	Amino acid transport and metabolism
F	63	2,5	Nucleotide transport and metabolism
H	69	2,7	Coenzyme transport and metabolism
I	57	2,2	Lipid transport and metabolism
P	107	4,2	Inorganic ion transport and metabolism
Q	12	0,5	Secondary metabolites biosynthesis, transport and catabolism
R	0	0	General function prediction only
S	1025	40,4	Function unknown
-	94	3,7	Not in COGs

^a^the total is based on the number of protein coding genes in the annotated genome

## Insights from the genome sequence

In addition to the automatic genome annotation by PGAAP, KAAS [49] was used to analyse metabolic features of the strain LP1^T^. The *L. profundi* LP1^T^ genome encodes for all central carbohydrate metabolic pathways (Additional file 1: Table S1); Embden-Meyerhof-Parnas pathway, gluconeogenesis and the TCA cycle. The genome contains genes for the non-oxidative branch of the pentose-phosphate-pathway, however misses the genes for the oxidative branch. Genes for the glyoxylate shunt of the TCA cycle are not present. The key enzyme ATP citrate lyase (EC 2.3.3.8) of the rTCA was not found. Besides the pyruvate dehydrogenase complex, a pyruvate:ferredoxin oxidoreductase (Lupro_00440) was identified, which may also catalyse the reverse reaction from acetyl-CoA to pyruvate. *L. profundi* LP1^T^ harbours the gene for phosphoenolpyruvate carboxylase (Lupro_02180), which may convert phosphoenolpyruvate into oxaloacetate, fixing CO_2_ in an anaplerotic reaction [22, 23]. Genes for energy generation via oxidative phosphorylation were identified (Additional file 1: Table S1). The major components comprise the NADH-dehydrogenase complex I, the succinate dehydrogenase/fumarate reductase complex II, a variety of quinone, and cytochrome c terminal oxidoreductases. Energy generation in form of ATP could be provided by the encoded F_0_F_1_-type ATP synthase. In addition to a H(+)-translocating NADH-dehydrogenase complex, a Na(+)-translocating NADH-quinone reductase is encoded in the genome, a feature common in marine bacteria [50]. Different aerobic terminal oxidoreductases could be identified, such as cytochrome c oxidases, cytochrome bo3 ubiquinol oxidase, cbb3-type cytochrome c oxidase and quinol oxidizing cytochrome bd-I terminal oxidase.

All genes for the complete denitrification pathway, from nitrate to nitrogen (NapAB, NirS, NorBC, NosZ), were identified in the strain LP1^T^ (Additional file 1: Table S1). Nitrate reduction to nitrite was confirmed in growth experiments under aerobic and microaerophilic conditions, while anaerobic growth using nitrate as the sole electron acceptor was not observed [1]. One ammonium transporter (Lupro_05500) was detected for ammonia assimilation. Ammonia can be fixed indirectly by glutamine synthetase and GOGAT, or directly by NADP-dependent glutamate dehydrogenase forming glutamate. Two different forms of GOGAT were identified, a NADPH dependent and a ferredoxin-dependent. The absence of genes encoding for urease is in concordance with the phenotypic characterization [1]. Genes for oxidation of sulphide, SQR, polysulfide reductase and sulphate permease, were identified in the genome of *L. profundi* LP1^T^ (Additional file 1: Table S1). However, growth of *L. profundi* LP1^T^ was not stimulated in the presence of thiosulfate under microaerobic or anaerobic conditions [1]. The presence of a SQR could also be an adaptation to the elevated concentration of sulphide emitted from the vent fluids at LCVF, rather than growth.

### Potential role of *Lutibacter profundi* LP1^T^ as complex organic compound degrader in the deep-sea biofilm

The organotroph *L. profundi* LP1^T^ was isolated from a microbial biofilm where a *Bacteroidetes* population was found attached to filamentous *Epsilonproteobacteria* producing a sugar biopolymer resembling chitin or cellulose [32]. The dbCAN analysis detected 101 proteins exhibiting one or more functional activities within CAZy [51, 52]. GTs (45) are mainly represented, followed by GHs (24), CEs (24), PLs (1) and CBMs (7). Ten GH families (Additional file 2: Table S2) are found in the genome, whereof GH13 and GH74 represent half of the enzymes. Diverse GH13 hydrolases, partially located in a Sus, cluster enable the bacterium to utilize starch. Characterization of *L. profundi* LP1^T^ has shown its ability to grow on starch and sucrose as the single C-source [1]. The strain also has the ability to catabolise monosaccharides such as mannose-6P, fructose-6P and glucose, as well as the disaccharides maltose, sucrose and trehalose. A sugar kinase (Lupro_07775) could activate monosaccharides such as mannose and fructose by phosphorylation [53]. The ability of the strain LP1^T^ to degrade starch [1] was supported by the presence of a Sus (Lupro_12175-Lupro_12250). Additional, two other SusD proteins (Lupro_05305 and Lupro_02600) and three signal peptide containing proteins (Lupro_10330, Lurpo_05115 and Lurpo_05135), described as ‘Starch-binding associating with outer membrane’, were found in the genome adjacent to TonB-linked outer membrane transporter proteins. These proteins harbour a SusD-like_2 domain and facilitate extracellular starch-binding, while being associated to the outer membrane with an N-terminal lipid tail [54]. For the polysaccharide degradation specialist *Bacteroides thetaiotaomicron*, SusC and SusD alone account for ~60% of the polysaccharide-degrading ability [55]. Furthermore, a gene for a bacterial glycogen synthase (Lupro_08100) was found in the *L. profundi* LP1^T^ genome that would allow energy conservation in form of glycogen.

Conserved domain [48] prediction revealed a possible neuraminidase/sialidase function for the GH74 hydrolases, alongside with a general function for β-1,4-linked glucan hydrolase activity for this family based on CAZypedia [56]. Bacterial sialidases are involved in the removal of sialic acid from various glycoconjugates [57] and are so far classified in the GH families 33 and 58 [58]. However, most GH74 hydrolases exhibit specificities towards xyloglucans and/or xyloglucan-oligosaccharides found in plant cell walls [59]. Either way, these predicted enzymes might be involved in the degradation of oligosaccharides. GHs, belonging to GH3, GH20, GH23, GH73 and GH109, can be linked to modification/degradation of cell wall components such as peptidoglycan, glycoproteins and lipopolysaccharide. Two peptidoglycan-modifying enzymes, Lupro_08335 (GH23) and Lupro_11420 (GH73), are supplemented with a CBM family 50 mediating the binding to N-acetylglucosamine residues [60]. Various outer membrane proteins containing SusC domains and TonB-dependent receptors enable oligosaccharide import into the periplasm and from there through sodium/glucose co-transporter and L-fucose-proton symporter to the cytosol. Compared to carbohydrate active enzymes, *L. profundi* LP1^T^ harbours a larger number of proteases. Positive degradation of gelatine and casein on agar plates was observed for *L. profundi* LP1^T^ [1]. 131 gene-encoding sequences were assigned to 51 MEROPS peptidase families, mostly metallo- and serine proteases (Additional file 3: Table S3), whereof 27 contained a signal peptide. From marine sedimentary bacteria the majority of extracellular peptidases have been identified as serine- and metalloproteases [61, 62]. The peptidase families C26, M01, M14, M20, M23, S09, S12, S33, and S41 were found more frequently than others. The amount of M01 and S09 peptidases are similar to the deep-sea *Bacteroidetes* *Zunongwangia profunda* SM-A87, as well as the high number of peptidase genes from the families M01, M23, S09, and S41 [62]. Secreted M01 aminopeptidase in *Z. profunda* SM-A87 has been proposed as a response to HMW dissolved organic nitrogen degradation, whereas the prolyl oligopeptidases of family S09 specifically hydrolyse oligopeptides shorter than 30 residues [62].

For the accessibility of nutrition deriving from HMW organic matter, hydrolytic enzymes need to be exported across the cell envelope into the extracellular environment. In total, 71 genes encoding for proteins of the double-membrane-spanning secretion systems type I (T1SS), and efflux pumps are incorporated in the *L. profundi* LP1^T^ genome (Additional file 4: Table S4). Both systems are often associated with nutrition acquisition and antimicrobial resistance mechanisms [63]. The T1SS use ABC transporters for substrate translocation across the cytoplasma membrane, whereas efflux pumps use Na+/H+ drug antiports or the proton-motive force [64]. 32 proteins were associated with ABC transport across the inner membrane. Whereas 6 RND transporters, 13 major facility transporters and 7 multidrug and toxic compound extrusion family proteins was identified as efflux pumps. In total, 10 outer membrane channel proteins TolC were identified, transporting substrate from the periplasm across the outer membrane in both systems [64].

Six genes related to the curli biogenesis system (Lupro_11990-Lupro_12015) were found. Curli fibers produced by the curli biogenesis system have shown to be involved in adhesion to surfaces, cell aggregation and biofilm formation [65]. Cell morphology changes were observed in *L. profundi* LP1^T^ into filamentous rods and cell aggregation under sub-optimal cultivation condition, such as the presence of ampicillin, non-optimal temperatures, unfavourable carbon source or extended growth periods above one week (Fig. 2b) [1]. The abilities to aggregate or produce biofilms are also beneficial, and perhaps vital for *L. profundi* LP1^T^ to survive the fluctuating chemical and physical conditions of the deep-sea hydrothermal vent system. A variety of protein domains involved in adhesion was identified using NCBI Batch web CD-Search Tool [48]. In total 60 ORFs were revealed from the genome, containing adhesion domains such as FN3, TSP_3, vWA, CBM’s, LamG, PKD, among others (Additional file 5: Table S5). Many bacterial species also produce extracellular polysaccharides that are able to promote adhesion [66]. In the genome of *L. profundi* LP1^T^ three genes encoding for poly-β-1,6-N-acetyl-D-glucosamine synthase/GT family 2 (Lupro_00610, Lupro_00765, Lupro_09885) and a potential polysaccharide deacetylase gene (Lupro_10410) were found, which may enable the bacteria to produce poly-β-1,6-N-acetyl-D-glucosamine (PGA). The homopolymer PGA mediates cell-to-cell and cell-to-surface adhesion in biofilms in *E. coli* and has effects on diverse host-microbe interactions [67]. The O-antigen of lipopolysaccharides can mediate attachment to host surfaces and biofilm formation [68, 69]. The strain LP1^T^ comprises extracellular polysaccharide gene clusters containing several glycosyl transferases, besides genes encoding for lipid A synthesis, which may also attribute towards cell adhesion and biofilm formation.

Many members of the *Bacteroidetes* are able to glide along surfaces in search for nutrition or as response to environmental stimuli [21, 70]. Blast analysis of the *L. profundi* LP1^T^ genome revealed 17 protein-encoding genes involved in gliding motility (Additional file 6: Table S6). However, no gliding motility has been observed for *L. profundi* LP1^T^ [1]. *Bacteroidetes* strains, such as the non-motile oral pathogen *Porphyromonas gingivalis* or F. johnsoniae use the gliding motility apparatus in addition for secretion of extracellular enzymes participating in accessing nutrition or serve as virulence factors [71, 72]. The gliding motility apparatus has been suggested to refer to PorSS as the type IX secretion system (T9SSs) [70]. In the genome of strain LP1^T^, 17 proteins were found containing a Por_Secre_tail domain, which is responsible for translocation of proteins across the outer membrane via PorSS [73]. Amongst these proteins are adhesins, proteases, an endonuclease, an α-amylase and a putative sialidase. Therefore the PorSS may not only add to the transportation system of *L. profundi* LP1^T^, but also enhances its hydrolytic capacity.

## Conclusions

The genome of *Lutibacter profundi* LP1^T^ comprises a single chromosome of 2,966 Mbp, smaller compared to other marine *Bacteroidetes* [21, 62, 74]. A reduced genome, a range of transporter systems and metabolic features indicate a highly specialized organism toward a life in a deep-sea hydrothermal vent biofilm.

*L. profundi* LP1^T^ originated from a biofilm attached to the outer surface of a deep-sea hydrothermal chimney. The mat consisted of long recalcitrant sugar polymers produced by the *Epsilonproteobacteria* *Sulfurovum* with *Bacteroidetes* attached along the filament surface [32]. As organotrophs, *Flavobacteriaceae* have been linked to HMW organic matter degradation such as polysaccharides and proteins. *L. profundi* LP1^T^ features a small selected arsenal of 24 GHs, which is rather a minor amount compared to other members of the family [21, 74], nevertheless it offers the possibility to hydrolyse α-glucosidic poly- and oligosaccharides, peptidoglycans and β-glycans. The utilisation of starch and sucrose was confirmed by the presence of a Sus cluster. Together with the large number of proteases strain LP1^T^ seems predestined to utilize complex organic matter efficiently, derived from a microbial biofilm. Diverse TonB-dependent receptors located close to glycoside hydrolases and proteases, as well as sodium/glucose cotransporter, amino acid permeases and transporter confirm the organotrophic life style. *L. profundi* LP1^T^ contains a set of genes for gliding motility, which is common in *Bacteroidetes* [70], and may allow the strain to move in the biofilm. Furthermore, the gliding motility apparatus seems to add to the transportation system of *L. profundi* LP1^T^, by exporting Por secretion signal containing proteins such as protease, endonuclease, amylase, putative sialidase or proteins with adhesive properties, which contributes to accessibility of nutrition’s for the bacteria. *L. profundi* LP1^T^ can mediate attachment to surfaces via a multitude of adhesins and extracellular polysaccharides and thereby may contribute to the biofilm generation. The presence of various cytochrome c oxidases with different oxygen affinities enables the bacteria to thrive in microaerophilic to aereophilic conditions, like they are present in biofilms or hydrothermal environments influenced by fluctuation of hydrothermal fluids mixed with sea water. The microaerobic life style is further indicated by diverse ferredoxin utilizing enzymes. The complete pathway for denitrification is present in *L. profundi* LP1^T^ in addition to oxygen respiration and the activity of nitrate reduction to nitrite has been confirmed under microaerobic conditions, although it did not enhance the growth [1]. Furthermore, SQR involved in the sulphur metabolisms may play an important role in sulphide detoxification in an environment with high sulphide concentration.

## Additional files

Additional file 1: Table S1. Central metabolism of *Lutibacter profundi* LP1^T^. (XLSX 37 kb)
Additional file 2: Table S2. Glycoside hydrolases of *Lutibacter profundi* LP1^T^ revealed by dbCAN analyses. different export signals are SP: signal peptid, PorSS: Por_Secre_tail from type IX secretion system. (XLSX 47 kb)
Additional file 3: Table S3. Proteases of *Lutibacter profundi* LP1^T^ revealed by MEROPS analyses, different export signals are SP: signal peptid, PorSS: Por_Secre_tail from type IX secretion system. (XLSX 44 kb)
Additional file 4: Table S4. Secretion systems of *Lutibacter profundi* LP1^T^. (XLSX 47 kb)
Additional file 5: Table S5. Genes and domains with a potential role in adhesion found in the *L. profundi* LP1T genome. (XLSX 38 kb)
Additional file 6: Table S6. CDS for gliding motility. (XLSX 38 kb)

## Abbreviations

AMOR: Arctic mid-Ocean ridge
CAZy: Carbohydrate active enzyme families
CBMs: Carbohydrate binding modules
CEs: Carbohydrate esterases
DPG: Diphosphatidylglycerol
GHs: Glycoside hydrolases
GOGAT: Glutamate-oxoglutarate aminotransferase
GTs: Glycosyl transferases
HMW: High-molecular weight
KAAS: KEGG automatic annotation server
LCVF: Loki’s castle vent field
NSC: Norwegian sequencing centre
PE: Phosphatidylethanolamine
PGAAP: Prokaryotic Genome annotation pipeline
PLs: Carbohydrate esterases
RND: Resistance, Nodulation and cell Division
SQR: Sulphide:quinone reductase
Sus: Starch utilization cluster
